# Intestinal Metabolome for Diagnosing and Prognosing Autism Spectrum Disorder in Children: A Systematic Review

**DOI:** 10.3390/metabo15040213

**Published:** 2025-03-21

**Authors:** Andrés Suárez-Jaramillo, Sara G. Cifuentes, Manuel Baldeón, Paúl Cárdenas

**Affiliations:** 1 Institute of Microbiology, Universidad San Francisco de Quito, Quito 170901, Ecuador; asuarezj@estud.usfq.edu.ec (A.S.-J.); gcifuentes@asig.com.ec (S.G.C.); 2 Facultad de Ciencias Médicas de la Salud y de la Vida, Universidad Internacional del Ecuador, Quito 170411, Ecuador; mabaldeonti@uide.edu.ec; 3 Unidad de Investigación Clínica, Hospital Metropolitano de Quito, Quito 170521, Ecuador

**Keywords:** autism spectrum disorder, intestinal metabolome, diagnosis, prognosis, neurodevelopmental scales

## Abstract

**Background/Objectives**: Currently, the diagnosis of autism spectrum disorder (ASD) relies on behavioral observations, frequently causing delays in early identification. Prognostic markers are essential for customizing therapy and monitoring progress. However, there are currently no recognized biomarkers for ASD. The current systematic review aims to analyze studies on the intestinal metabolome in children (both autistic and non-autistic) to identify potential metabolites for diagnostic and prognostic purposes. **Methods**: We searched Medline, Scopus, Embase, and Web of Science for relevant publications. **Results**: We identified 11 studies examining the gut metabolome that distinguished between autistic and non-autistic children. These studies also revealed connections between gut metabolites, developmental scores, and symptoms. The substances identified were associated with metabolic pathways such as amino acids, vitamins, lipids, oxidative stress, glycans, xenobiotics, and nucleotides. **Conclusions**: These findings suggest metabolic changes that may be linked to the causes or development of autism. Although these observations came from a few reports, only high-quality studies were included in this review. Further research is essential to confirm the identified substances as biomarkers.

## 1. Introduction

Autism spectrum disorder (ASD) is an early childhood neurodevelopmental disorder characterized by deficits in social communication and social interactions across various contexts. It is also accompanied by restricted, repetitive, and stereotyped patterns of behavior, interest, or activities [1]. The prevalence of ASD has increased from 1990 to 2019, with a median estimated at 100/10,000. Therefore, it is considered a global public health challenge [2,3]. Early intervention of autistic children improves the efficacy of behavioral therapies [4]. However, an ASD diagnosis is based on the observation of behaviors that are difficult to identify in the first years of life. The average age of diagnosis is between 3 and 4 years, with many individuals not being diagnosed until puberty [4,5,6]. Accurate prognosis is necessary to know the expected future outcomes of the child and to individualize treatment [7]. Therefore, new strategies are needed to diagnose and determine the prognosis of children with ASD. Biomarkers are considered quantitative characteristics of normal biological processes, diseases, or treatment responses and are possible alternatives to diagnose and follow-up individuals with ASD [8]. However, no proven biomarkers currently exist that can be used as diagnostic or prognostic indicators in ASD [9,10].

The causes of ASD are not fully understood, but they are likely multifactorial. The interaction between environmental and genetic factors is probably involved in ASD etiology [11]. It has been postulated that alterations of the microbiota–gut–brain axis could be associated with ASD [12]. The microbiota–gut–brain axis consists of a bidirectional communication between the gut and brain mediated by the nervous, immune, and endocrine systems. In this communication, microbiota derived molecules such as short-chain fatty acids, lipopolysaccharides, tryptophan, and glutamate derivates also intervene [13]. Gut microbiota dysbiosis could alter the gut metabolome (metabolites produced by intestinal microorganisms and the host). The altered level of many metabolites can modulate the microbiota–gut–brain axis, making alterations in brain function [11,13,14]. Fecal metabolites can be linked to leaky gut, intestinal inflammatory processes, and gastrointestinal symptomatology, also associated with ASD [15,16]. As a result, gut-derived metabolites could be used as diagnostic and prognostic markers in autistic children. In clinical practice, biomarkers must be easy to obtain, non-invasive, and present in a peripheral fluid or tissue. Molecules present in fecal samples meet these criteria. This facilitates using fecal metabolites in clinical practice, not only in the initial diagnosis, but also in the follow-up, which requires multiple sampling [17,18].

In this systematic review, we searched Medline, Embase, Scopus, and the Web of Science to find studies that analyzed the intestinal metabolome and its relationships with the prognosis and diagnosis of ASD. With the obtained information, we aimed to identify metabolites that could contribute to the diagnosis and prognosis of ASD.

## 2. Materials and Methods

The systematic review adhered to the Preferred Reporting Items for Systematic Reviews and Meta-analysis (PRISMA) guidelines. The protocol was registered on the PROSPERO database (CRD42023406088).

### 2.1. Eligibility Criteria

We included cross-sectional, cohort, case–control, and clinical trial studies. All studies included participants under 18 years of age. Children with ASD were required to have a diagnosis made by a specialist or based on accepted diagnostic criteria. For studies focused on ASD diagnosis, we compared the intestinal metabolome of children with ASD to that of non-autistic children. For prognosis-related studies, comparisons were made either between different groups of children with ASD or between ASD and non-autistic children. Studies involving ASD children with genetic syndromes were excluded.

### 2.2. Study Selection and Data Extraction

**Figure 1:**
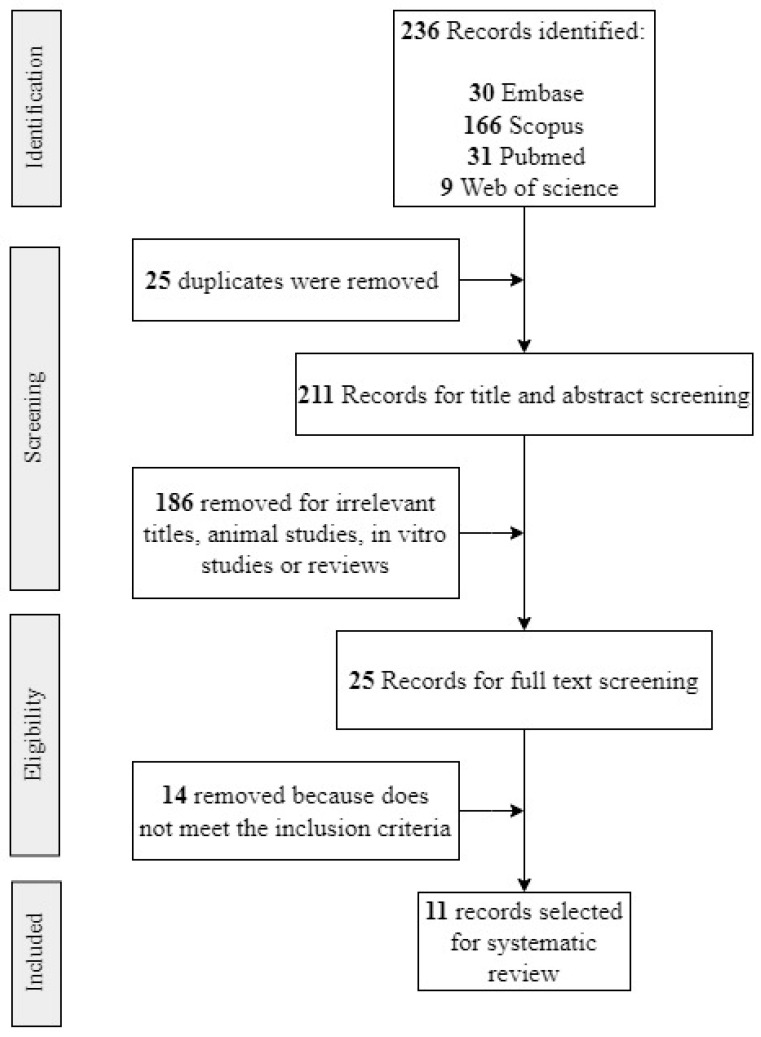
Flowchart for study retrieval and selection.

A comprehensive systematic search was conducted in May 2023 using the Embase, Web of Science, Scopus, and Medline databases. We included peer-reviewed articles published in English or Spanish, with no restrictions on the publication year. The complete search strategies are detailed in Table 1. The information obtained was downloaded to spreadsheets, and duplicates were deleted (Figure 1). Using the eligibility criteria, two independent reviewers (A.S.J and S.C) analyzed the titles and abstracts. After the initial review, the full text of the selected papers was screened to determine eligible studies. A third reviewer (P.C.) was consulted in the case of divergences. Two independent reviewers (A.S.J and S.C) extracted data using a data extraction form. In the selected papers, outcomes were measured using the odds ratio (OR), relative risk (RR), mean differences, PERMANOVA, MANOVA, partial least-squares discriminant analysis (PLS-DA), orthogonal partial least squares-discriminant analysis (OPLS-DA), principal coordinate analysis (PCoA), principal component analysis (PCA), correlation statistics (Spearman, Pearson, Kendall), or other statistical methods to find a correlation or association between the metabolomics and diagnosis/prognosis.

### 2.3. Risk of Bias Assessment

Two independent (A.S.J and SC) reviewers were involved in the risk of bias analysis of the selected papers. Any disagreements were resolved by consultation with a third reviewer (P.C). The modified instrument developed by Van’t Hof et al. was used for the risk of bias assessment of the observational studies [19]. This tool provides a numerical value that represents the risk of bias. The highest possible score is 9, representing the highest risk of bias. ROBINS-I [20] was used in non-randomized trials. This tool classifies bias into the following categories: low risk, moderate risk, serious risk, or critical risk.

## 3. Results

### 3.1. Study Selection

A total of 236 results were retrieved from the systematic search in Medline, Web of Science, Embase, and Scopus (Figure 1). After removing 25 duplicates, 211 unique studies remained. After screening the titles and abstracts, 25 full-text articles were assessed for eligibility. Ultimately, 11 studies met the inclusion criteria and were included in this systematic review (Figure 1). The reasons for excluding studies are detailed in Supplementary Table S1.

### 3.2. Characteristics of Included Studies

The 11 studies included were published between 2019 and 2023 and were written in English. A summary of the selected studies is provided in Table 2. Two studies were clinical trials, and nine were observational. Most papers were from the United States (4) and China (4). Additionally, one paper was from Tunisia, one from Italy, and one from Australia. Sample sizes were from 24 to 286 participants (autistic and non-autistic children). Participants were 16 years old or less in the study period. Various diagnostic methods for ASD were used including the Diagnostic and Statistical Manual of Mental Disorders, Fifth Edition (DSM-5), the Autism Diagnostic Observation Schedule, Second Edition (ADOS-2), the Autism Diagnostic Interview-Revised (ADI-R), and specialist evaluations.

### 3.3. Intestinal Metabolome and ASD Diagnosis

The studies included in this review identified several intestinal metabolites involved in different metabolic pathways that could potentially be used for ASD diagnosis. These metabolites are related to pathways commonly associated with ASD such as amino acid metabolism, vitamin metabolism, lipid metabolism, oxidative stress, glycans, xenobiotics, and nucleotides. A detailed list of these metabolites and their associations is provided in Table 3.

**Figure 2:**
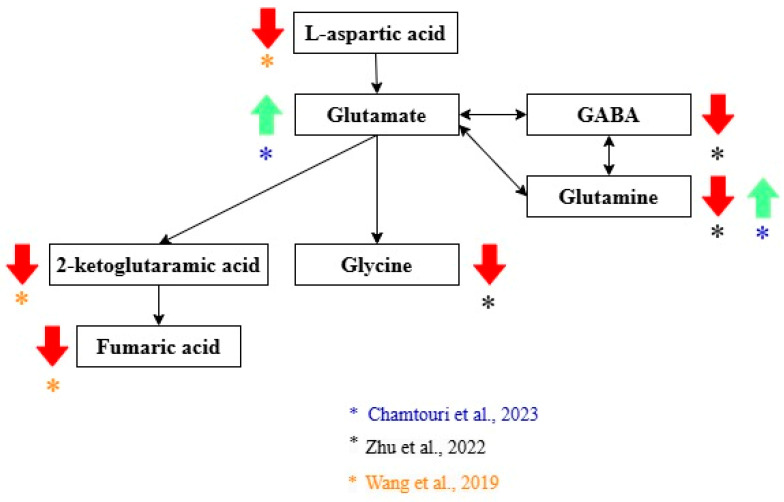
Glutamate metabolites related to the diagnosis of ASD. Green arrows show metabolites that increase, and red arrows show metabolites that decrease in ASD. Black arrows indicate the direction of the pathway. The color of the asterisks represents the author that found the indicated metabolic changes [21,22,23,24].

Three studies highlighted alterations in glutamate metabolism in autistic children compared with non-autistic controls [21,22,23] (Figure 2). L-aspartic acid, 2-keto-glutaramic acid, and fumaric acid were significantly lower in ASD children compared with children without autism [22]. Wang et al. performed a validation of 2-keto-glutaramic acid as a biomarker of ASD by performing two separate experiments with two different samples comparing the gut metabolome of non-autistic and children with ASD [22]. A decrease in glycine, GABA, and glutamine levels was observed in ASD [21]. However, Chamtouri et al. found significantly higher levels of glutamine and glutamate in the ASD group. This difference was more pronounced in children 4 to 7 years old [23].

**Figure 3:**
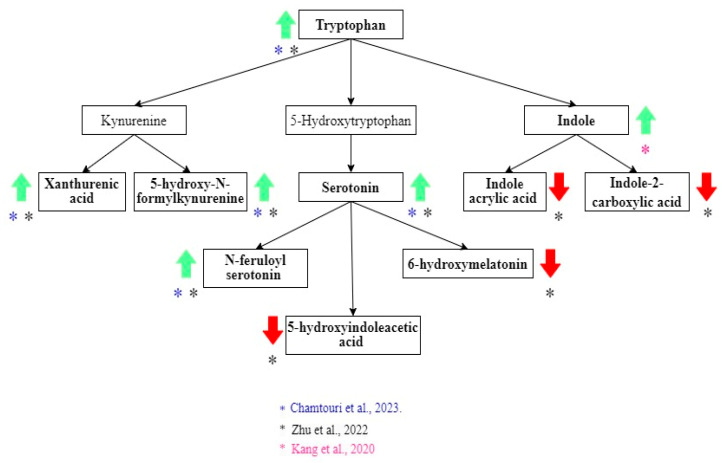
Tryptophan metabolites related to the diagnosis of ASD. Green arrows show metabolites that increase, and red arrows show metabolites that decrease in ASD. Black arrows indicate the direction of the pathway. The color of the asterisks represents the author that found the indicated metabolic changes [21,23,24,25].

Alterations in tryptophan metabolism were also common in ASD children [21,23] (Figure 3). The increase in tryptophan, xanthurenic acid, 5-hydroxy-N-formylkynurenine, serotonin, and N-feruloyl serotonin was associated with ASD diagnosis [21,23]. Decreased levels of 5-hydroxy indole acetic acid and 6-hydroxy melatonin were described in the ASD group [21]. Indole had a higher concentration in the ASD group. However, the difference between the non-autistic group was not statistically significant when the correction for multiple hypotheses was applied [25]. In contrast, Zhu et al. found that two indole derivates (indole acrylic acid and indole-2-carboxylic acid) were lower in ASD children [21].

**Figure 4:**
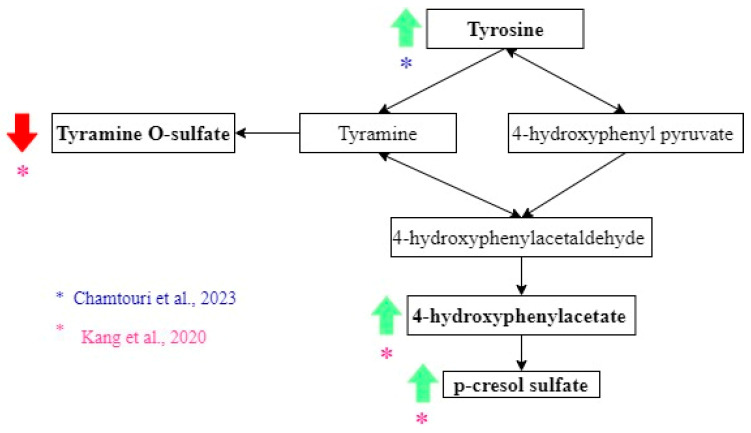
Tyrosine metabolites related to the diagnosis of ASD. Green arrows show metabolites that increase, and red arrows show metabolites that decrease in ASD. Black arrows indicate the direction of the pathway. The color of the asterisks represents the author that found the indicated metabolic changes [23,24,25,26,27,28].

Kang et al. found that tyrosine derivatives had different abundances between the ASD and non-autistic groups (Figure 4). The 4-hydroxyphenylacetate and p-cresol sulfate levels had higher concentrations in feces from the ASD group [25], while tyramine O-sulfate was lower in feces from the ASD group. However, these differences were insignificant when the correction for multiple hypotheses was applied [25]. Nevertheless, significantly higher concentrations of tyrosine were measured in ASD children, according to another study [23].

Zhu et al. encountered alterations in substances related to vitamins in children with ASD. Vitamin A precursors and intermediates, such as 4′-apo-beta-carotenal and retinal, were increased in ASD children; conversely, retinol was decreased. Reduced levels of thiamine pyrophosphate, riboflavin, phosphopantothenic acid (vitamin B5 derivative), pyridoxamine, and vitamin C were noted in ASD children [21]. Dihydrofolate and 5-methyltetrahydrofolate, two folic acid substances, were also lower in ASD children [21].

**Table 2:** Characteristics of selected studies.

Author(s)	Title	Year	Metabolomics Method	Number of Participants	Age	Sex (M/F)	Country	Recruitment	ASD Diagnosis Method
Needham et al. [4]	Plasma and fecal metabolite profiles in autism spectrum disorder	2021	UHPLC-MS/MS	Plasma samples. **ASD:** 130, **NA:** 101. Fecal samples in a subset. **ASD:** 57, **NA:** 40	ASD and NA: 3–12 years old	Not clear information. **ASD:** 48/7. **NA:** 36/3	United States	Samples were collected in the MIND Institute, University of California, Davis. ASD diagnosis was confirmed by trained staff. NA participants were screened using the Social Communication Questionnaire and scored within the typical range. An additional evaluation to determine gastrointestinal (GI) symptoms was completed by 97 participants who also provided stool samples. GI status was determined using the GI symptom scale, based on Rome III Diagnostic Questionnaire on Pediatric Functional Gastrointestinal Disorders.	ADOS2, ADI-R, DSM5
Zhu et al. [21]	Alterations in gut vitamin and amino acid metabolism are associated with symptoms and neurodevelopment in children with autism spectrum disorder	2022	LC–MS/MS	**ASD:** 120. **NA:** 60	**ASD and NA:** 2 to 6 years.	**ASD:** 99/21. **NA:** 39/21	China	The inclusion criteria were a diagnosis of ASD made by a developmental pediatrician. The exclusion criteria were other developmental disorders, psychiatric or neurological diseases, genetic or metabolic illness, major physical disease, a recent history of infection, special diets, or probiotic/antibiotic use. NA children were healthy, and showed no sign of developmental disorders, psychiatric diseases, or GI symptoms.	DSM5, CARS
Wang et al. [22]	Alterations in gut glutamate metabolism associated with changes in gut microbiota composition in children with autism spectrum disorder	2019	LC-MS	**ASD:** 92 (43 for the initial analysis and 49 for validation); **NA:** 42 (31 for the initial analysis and 11 for validation).	**ASD and NA:** Less than 14 years old.	**ASD:** 36/7. **NA:** 17/14	China	ASD: Children younger than 14 years old and classified as “requiring very substantial support” were included. Exclusion criteria were: diagnosis of other mental illness, other neurodevelopmental condition, genetic metabolic disease, or severe neurological disease; history of brain injury; or history of other major physical illness. NA: Healthy children, with no mental illness, and age- and gender-matched with the patient group. Exclusion criteria were the same as for the ASD group. Signed informed consent was obtained from the parents of ASD and NA children.	DSM5
Chamtouri et al. [23]	An overview on fecal profiles of amino acids and related amino-derived compounds in children with autism spectrum disorder in Tunisia	2023	UHPLC	**ASD:** 28; **NA:** 46 (divided in two groups: 18 siblings of the participants and 28 children from the general population matched with autists by gender, age, socio-economic status, and geographic region).	**ASD and NA:** 4 to 10 years old	**ASD:** 22/6. **NA:** 27/19	Tunisia	Authors considered three groups: autistic children, siblings of autistic children and general population children. The last 2 are considered NA. NA children did not show signs of developmental disorders or psychiatric diseases. The exclusion criteria for the three groups were having suffered infections, antibiotics or anti-fungal treatment or having consumed probiotics and/or prebiotics in the previous month of recruitment, unbalanced or special diets, other neurological disorders not associated with autism, type 1 diabetes, genetic syndromes, celiac disease, food intolerance, or inflammatory bowel disease.	DSM5, ADOS2. ADI-R, CARS
Kang et al. [25]	Distinct fecal and plasma metabolites in children with autism spectrum disorders and their modulation after microbiota transfer therapy	2020	UHPLC-MS/MS	**ASD:** 18 **NA:** 20	**ASD and NA:** 7–16 years old	**ASD:** 16/2. **NA:** 18/2	United States	Participants were recruited primarily from the greater Phoenix, Arizona area. The study physician assessed eligibility criteria Once qualified, participants engaged in an initial 30-min meeting which included a general physical health examination by the study physician and discussion with a project member. Exclusion criteria included antibiotics use in the prior 6 months or probiotics use in the prior 3 months; dependence on tube feeding; severe GI problems that require immediate treatment; recent/scheduled surgeries; diagnosed as severely malnourished or underweight; and diagnosed with a single-gene disorder, major brain malformations, ulcerative colitis, Crohn’s disease, celiac disease, or eosinophilic esophagitis. None of the NA children had been diagnosed with mental disorders including ASD, attention-deficit hyperactivity disorder (ADHD), depression, or anxiety. None of the neurotypical children had first-degree relatives with ASD.	ADI-R
Jones et al. [29]	Changes to the gut microbiome in young children showing early behavioral signs of autism	2022	GC-MS	Not clear but a total of 24 children were enrolled Six of the 24 children provided stool samples one and two years post-baseline.	**ASD and NA:** 9–14 months at the beginning of the study	Change between time points of the study (A and B.) **ASD:** A:3/1, B: 8/3. **NA:** A: 5/0; B: 4/1	Australia	Included children showing early social-communication delays as determined by Social Attention and Communication Surveillance–Revised (SACS-R) 12-month checklist. Families were referred by community clinicians and invited to participate. The infant displayed at least 3 of 5 specified behaviors indicating a high likelihood of ASD as defined by the Social Attention and Communication Surveillance–Revised (SACS-R) 12-month checklist and the primary caregiver spoke sufficient English to participate in the intervention. Infant were excluded if they had a neurological condition and other developmental problems and/or the family did not intend to remain residents in the local area during the study.	ADOS2
Berding et al. [30]	Dietary patterns impact temporal dynamics of fecal microbiota composition in children with autism spectrum disorder	2020	GC	**ASD:** 26, **NA:** 32	**ASD and NA:** 2–7 years old	**ASD:** 19/7. **NA:** 19/13	United States	Participants were recruited in the Champaign-Urbana area. All subjects were free from functional digestive disorders, had not used antibiotics, probiotics or prebiotic in the 3 months prior to enrollment in the study, did not take any routine medications and did not follow any special diet (e.g., gluten-free/casein-free diet). Parents completed an online questionnaire, including questions regarding their child’s age, gender, mode of delivery, early feeding practices, nutritional supplement use, height and weight.	It is not clearly explained.
Deng et al. [31]	Gastrointestinal symptoms have a minor impact on autism spectrum disorder and associations with gut microbiota and short-chain fatty acids	2022	GC-MS	**ASD:** 45, **NA:** 45.	**ASD:** 5.95 ± 2.36 years old. **NA:** 6.13 ± 0.90 years old	**ASD:** 39/6. **NA:** 21/24	China	Participants were enrolled from a local kindergarten. Children were excluded from the study if physical, neurological, and behavioral tests were performed for each participant. Children who had previously been diagnosed with genetic conditions (such as tuberous sclerosis, fragile X syndrome, and Rett syndrome), had received antibiotics or probiotics within 1 month, or were suffering from trauma, tumors, or other serious nervous system diseases.	DSM5
Qureshi et al. [32]	Multivariate analysis of fecal metabolites from children with autism spectrum disorder and gastrointestinal symptoms before and after microbiota transfer therapy	2020	UHPLC-MS/MS	ASD: 18, NA: 20	ASD and NA: 7–16 years old	**ASD:** 16/2. **NA:** 18/2	United States	Participants were recruited primarily from the greater Phoenix, Arizona area. The study physician assessed eligibility criteria Once qualified, participants engaged in an initial 30-min meeting which included a general physical health examination by the study physician and discussion with a project member. Exclusion criteria included antibiotics use in the prior 6 months or probiotics use in the prior 3 months; dependence on tube feeding; severe GI problems that require immediate treatment; recent/scheduled surgeries; diagnosed as severely malnourished or underweight; and diagnosed with a single-gene disorder, major brain malformations, ulcerative colitis, Crohn’s disease, celiac disease, or eosinophilic esophagitis. None of the NA children had been diagnosed with mental disorders including ASD, attention-deficit hyperactivity disorder (ADHD), depression, or anxiety. None of the neurotypical children had first-degree relatives with ASD.	ADI-R
Dan et al. [33]	Altered gut microbial profile is associated with abnormal metabolism activity of autism spectrum disorder	2020	LC/MS and UHPLC-QE	**ASD:** 143 (metabolomics was made in 30 children); **NA:** 143.	**ASD and NA:** 2–13 years.	**ASD:** 127/16. **NA:** 130/13	China	All of the children underwent physical, neurological, and behavioral examinations. The exclusion criteria were diseases such as depressive disorder, cerebral palsy, schizophrenia, bipolar disorder, significant sensory impairment, and clinically significant inflammatory conditions. All participants had not taken antibiotics, probiotics, and prebiotics in the 3 months prior to the feces collection. None of the participants were on anti-inflammatory or antioxidant drugs.	DSM5
Laghi et al. [34]	Are fecal metabolome and microbiota profiles correlated with autism severity? A cross-sectional study on ASD preschoolers	2021	H-NMR	**ASD:** 80	**ASD:** 18 to 72 months. **NA:** There is not a neurotypical group.	**ASD:** 66/14. **NA:** There is not a neurotypical group.	Italy	Participants were recruited during a clinical trial on the efficacy of probiotic supplementation in ASD preschoolers. Participants must have a diagnosis of ASD made by a senior child psychiatrist with a specific expertise in ASD. Exclusion criteria: brain anomalies detected by magnetic resonance imaging, neurological syndromes or focal neurological signs, birth asphyxia, severe premature birth (≤28 gestational weeks) or perinatal injuries, epilepsy, significant sensory impairment (blindness, deafness), diagnosis of organic GI disorder (gastro-esophageal reflux, food allergies, Inflammatory bowel disease),diagnosis of celiac disease, special diet (gluten-free diet, casein-free diet, high-protein diet, ketogenic diet).	DSM5, ADOS2

GI: gastrointestinal; ASD: autism spectrum disorder; NA: non-autistic; LC–MS: liquid chromatography-mass spectrometry; LC–MS/MS: liquid chromatography-tandem mass spectrometry; UHPLC-QE: ultra-high performance liquid chromatography coupled with quadrupole electrospray ionization mass spectrometry; UHPLC: ultra-high performance liquid chromatography; UHPLC-MS/MS: ultra-high performance liquid chromatography-tandem mass spectroscopy; H-NMR: proton nuclear magnetic resonance; GC-MS: gas chromatography coupled with mass spectrometry; GC: gas chromatography.

**Table 3:** Diagnostic metabolites in ASD.

Author(s)	Association with Diagnosis
Needham et al. [4]	Higher in ASD: Nicotinamide Higher in NA: 9-HOTrE
Zhu et al. [21]	Higher in ASD: Xanthurenic acid, 5-hydroxy-N-formylkynurenine, 5-hydroxytryptophan (5-HTP), serotonin, N-feruloyl serotonin 4′-apo-beta-carotenal, b,e-carotene-3,3′-diol, retinal, homocysteine, and 8-hydroxy-deoxyguanosine. Higher in NA: 6-Hydroxymelatonin and 5-hydroxyindoleacetic acid, retinol levels, dihydrofolate (DHF), 5-methyltetrahydrofolate (5-MTHF), N-acetylcysteine (NAC), and S-aminoethyl-L-cysteine, thiamine pyrophosphate (TPP), riboflavin (vitamin B2), phosphopantothenic acid (vitamin B5 derivative), pyridoxamine (vitamin B6), vitamin C, agmatine, spermine, glutathione spermidine, glutamine, GABA, and glycine.
Wang et al. [22]	Higher in ASD: Taurocholic acid and chenodeoxycholic acid 3-sulfate. Higher in NA: 2-Keto-glutaramic Acid (validated as a potential biomarker), L-aspartic acid, fumaric acid, cortisol, epinephrine, benzaldehyde, and cinnamic acid.
Chamtouri et al. [23]	Higher in ASD: Total fecal amino acids, branched-chain amino acids (valine, leucine, isoleucine), aromatic amino acids (tyrosine, phenylalanine, tryptophan), aliphatic amino acids (alanine, glycine), glutamate, and glutamine were found in ASD children.
Kang et al. [25]	Higher in ASD: p-Cresol sulfate, 4-hydroxyphenylacetate, and indole. Higher in NA: Thyramine O-sulfate. However, these differences were not significant when the correction for multiple hypotheses was applied.
Jones et al. [29]	Higher in ASD: Acetic acid and total SCFAs (short-chain fatty acids). However non-significant differences.
Berding et al. [30]	Higher in ASD: Propionate, isobutyrate, valerate, and isovalerate. This was evaluated in children with two specific dietary patterns. DP1: characterized by vegetables, starchy vegetables, legumes, nuts and seeds, fruit, grains, juice and dairy. DP2: fried, protein and starchy foods, “kid’s meals”, condiments, and snacks.
Deng et al. [31]	Higher in ASD: Propionic acid, butyric acid, and valeric acid.
Qureshi et al. [32]	Higher in ASD + GI problems: Carnitine. Two groups of 5 metabolites OFM-A (imidazole propionate, hydroxyproline, theobromine, 2-hydroxy-3-methylvalerate, adenosine) and OFM-I (imidazole propionate, hydroxyproline, theobromine, 2-hydroxy-3-methylvalerate, Indole). Both OFM had 95% specificity and 94% sensitivity in distinguishing ASD + GI from neurotypicals. Multivariate statistical analysis showed that groups of metabolites are more useful to discriminate between groups.
Dan et al. [33]	Higher in ASD: Quinic acid, 3-dehydroquinate, Thr-Phe, desaminotyrosine, vanillactic acid, indole-3-carboxylic acid, hexanoic acid, 3-indoxyl-D-glucopyranoside, 2,5-dioxopentanoate, γ-glutamylglycine, phosphatidylcholine, D-4′-phosphopantothenate, pantothenic acid, 3-(uracil-1-yl)-L-alanine, 3-dehydrocarnitine, methylselenocysteine Se-oxide, deoxyinosine,1-methyladenosine, orotidine-5P, 2′-deoxyuridine. Higher in NA: Tyr-Leu, DL-P-hydroxyphenyl lactic acid, indoleacetaldehyde, imidazole-4-acetaldehyde, adenine, deoxyadenosine, 2′-deoxyguanosine.
Laghi et al. [34]	No metabolites associated with diagnosis

ASD: autism spectrum disorder; NA: non-autistic; GABA: Gamma-aminobutyric acid; Thr-Phe: L-threonyl-L-phenylalanine; Tyr-Leu: L-tyrosyl-L-leucine; OFM-A: optimized fecal models-adenosine; OFM-I: optimized fecal models-indole; ASD + GI: ASD children with gastrointestinal problems; 9-HOTrE: 9-hydroxy-10E,12Z,15Z-octadecatrienoic acid.

Additionally, the ASD group had lower levels of N-acetylcysteine and S-aminoethyl-L-cysteine and higher levels of homocysteine. These substances are part of the cysteine-methionine pathway and are related to folate metabolism [21]. Needham et al. determined by random forest that nicotinamide, a form of B3 vitamin, was a critical diagnostic metabolite with a higher concentration in the ASD group [4].

Fecal fatty acids are other possible metabolites that may be used to discriminate ASD and non-autistic children. Jones et al. found no significant differences in the total or individual short-chain fatty acid (SCFA) concentration; however, children in the ASD group tended to have higher acetic acid and total SCFA concentrations [29]. Higher levels of propionate, isobutyrate, valerate, isovalerate, and butyrate were identified in children with ASD, indicating a potential association with the diagnosis of ASD [30,31]. Needham et al. determined by random forest that one of the key metabolites for diagnosis was 9-HOTrE, a fatty acid with a lower concentration in the ASD group [4].

Oxidative stress has been associated with ASD [4,21,22]. Benzaldehyde, an aromatic compound with antimicrobial and antioxidant functions, was significantly lower in ASD children [22]. Arachidonic acid metabolites involved in oxidative stress and inflammation were different in the ASD and non-autistic groups [21]. Arachidic acid and 20-hydroxy-leukotriene E4 were increased in ASD children while leukotriene B4 and 5-trans prostaglandin F2b were decreased in the ASD group. Additionally, 8-hydroxy-deoxyguanosine, a marker of oxidative DNA damage, was significantly associated with ASD [21]. There is also a relationship between mitochondrial dysfunction and oxidative stress. Carnitine and short-chain acetylcarnitine (markers of mitochondrial function) were higher in children with ASD [4,32]. Conversely, polyamines (related to oxidative stress protection) were lower in children with ASD [21].

Combinations of many substances involved in different metabolic pathways may have a diagnosis potential [32,33]. Dan et al. found alterations in metabolites involved in lipids, glycan, vitamins, amino acids, xenobiotics, and nucleotides. The modifications in these metabolic pathways could result in neurotransmitter metabolism changes, especially in GABA, dopamine, histidine, and serotonin [33]. Qureshi et al. conducted a univariate analysis and found that individual fecal metabolites had reduced potential to differentiate ASD children with gastrointestinal problems (ASD + GI) and non-autistic children without gastrointestinal pathology [32]. However, multivariate statistical analysis showed that groups of metabolites (optimized fecal models, OFM) are more valuable in discriminating between groups. Two groups of five metabolites, OFM-A (imidazole propionate, hydroxyproline, theobromine, 2-hydroxy-3-methylvalerate, adenosine) and OFM-I (imidazole propionate, hydroxyproline, theobromine, 2-hydroxy-3-methylvalerate, Indole), were found to have 95% specificity and 94% sensitivity in distinguishing ASD + GI from children without autism [32].

### 3.4. Intestinal Metabolome and ASD Prognosis

In addition to their role as markers for the diagnosis of ASD, fecal concentrations of some metabolites may be useful in determining the prognosis of these individuals. Several studies have found that gut metabolite levels are linked to the behavior and development scales. The metabolites involved are related to amino acids, proteins, oxidative stress, vitamins, and fatty acids [4,21,23,25,29,31,32,33,34]. The complete details are shown in Table 4.

As indicated in Table 4, different metabolites could be used as predictors of the presence of gastrointestinal symptoms, behaviors, sleep disturbances, and overall developmental status. Additionally, microbiota transfer therapy (MTT) lowered the p-cresol sulfate levels to a concentration similar to those of non-autistic children (the result was significative according to an un-adjusted *p* value) [25]. Analyzing the OFM models (OFM-I and OFM-A), Qureshi et al. found that the average difference in the normalized metabolite levels between the ASD + GI and non-autistic groups decreased significantly (by 82–88%) after MTT [32]. This suggests that some metabolites could be used to monitor the treatment with MTT. However, to define clinical applicability, more studies with larger samples and longer follow-up times are required.

### 3.5. Risk of Bias

In the present study, a modified instrument developed by Van’t Hof to evaluate the risk of bias was applied to the six observational studies. The lowest bias score was three, and the highest was five (maximum score of nine). A small sample was noted in six studies. In one study, the diagnosis method of ASD was not clearly explained. One study considered all relevant confounders in the analysis (Table 5). Two non-randomized clinical trials were selected and analyzed using the ROBINS-I tool. Both studies had a low risk of bias (Table 6).

## 4. Discussion

The identification of biological markers for the diagnosis and prognosis of diseases has a very important role in clinical practice [8,17]. The identification of disease biomarkers involves understanding the normal and pathological function of tissues, organs, and systems [8,17]. Recent studies of the metabolites of the intestinal microbiota and their relationship with health and disease states have demonstrated their potential usefulness in clinical practice [35]. This systematic review identified several fecal metabolites that may serve as potential diagnostic and prognostic markers for ASD. The metabolites span various metabolic pathways including amino acids, vitamins, lipids, oxidative stress markers, glycans, xenobiotics, and nucleotides. These findings suggest that metabolic dysregulation in these pathways could be associated with the pathophysiology of ASD. Additionally, the identified metabolites showed associations with the neurodevelopmental scores and symptoms, indicating their potential use in understanding ASD severity and prognosis.

Alterations in amino acids and their metabolic pathways have been reported in different studies. Two of the amino acids that may be associated with ASD are glutamate and GABA, which are related to the excitatory/inhibitory (E/I) imbalance hypothesis [36]. Imbalances in glutamate (primary excitatory neurotransmitter) and GABA (main inhibitory neurotransmitter) in different parts of the brain may explain the pathophysiology of autism [36,37]. For example, lower glutamate signals in magnetic resonance spectroscopy were found in the anterior cingulate cortex and cerebellum of autistic children compared with non-autistic [22,37]. Glutamate alterations are probably related to alterations in the gut microbiota [22]. Wang et al. found that a lower abundance of *Bacteroides vulgatus* and *Campylobacter jejuni* was correlated with lower levels of the D-glutamine and D-glutamate metabolic pathways [22]. 2-Keto-glutaramic acid, a product of glutamate metabolism, was validated as a diagnosis biomarker in a second experiment conducted with an additional sample of ASD and NA children [22].

Previous studies have described tryptophan metabolism alterations in autism including low tryptophan and high serotonin concentrations in the blood [38,39]. *Streptococcus*, *Lactobacillus*, *Klebsiella*, and *Escherichia coli* can synthesize serotonin from tryptophan via tryptophan synthetase, showing the relationship between the gut microbiome and serotonin metabolism [40]. The kynurenine pathway is one of the metabolic routes of tryptophan that has been related to ASD. Activation in this pathway can increase neurotoxic, excitotoxic, and immune suppressive mediators [41]. Like previous studies, in this systematic review, we found evidence of higher levels of components of the kynurenine pathway in children with ASD: the xanthurenic acid and 5-hydroxy-N-formylkynurenine levels [21]. However, more studies are needed to understand the exact role of kynurenine and its derivatives, since not all components of this pathway are deleterious; for example, kynurenic acid is neuroprotective [41]. Hyperserotonemia and melatonin deficit are possibly involved in ASD [21,38]. Considering that 95% of the body’s serotonin is produced in the enterochromaffin cells of the intestine, higher feces concentrations could reach the blood and have systemic effects [42]. Our systematic review found a negative correlation between the fecal serotonin levels and neurodevelopmental score [21]. This shows the possible relationship between intestinal serotonin and the outcomes of autistic children. Zhu et al. found that in autistic children, lower levels of 6-hydroxy melatonin, a product of melatonin metabolism, were used in clinical practice to determine the melatonin levels. In this regard, serum melatonin levels affect sleeping patterns, immune, and gastrointestinal functions [43]. At the intestinal level, melatonin is involved in peristalsis and inflammation regulation [43].

P-cresol is produced by the bacteria of the families *Clostridiaceae*, *Lachnospiraceae*, and *Ruminococcaceae* from tyrosine and phenylalanine [44]. Approximately 95% of p-cresol is metabolized to p-cresol sulfate by sulfation in the liver [25,44]. Although the mechanisms of action of p-cresol are not completely clear, they probably include neuron membrane depolarization, decreased Na^+^-K^+^ ATPase activity, reduced dopamine conversion to norepinephrine (inhibition of dopamine-β-hydroxylase), oxidative stress, and microglia dysfunction [25,44,45]. Kang et al. found a non-significant elevation (using multiple-hypotheses testing) in the fecal level of p-cresol sulfate in autistic children compared with non-autistic and observed a decrease in levels after microbiota transfer therapy [25]. This shows the potential utility of this substance in diagnosis and prognosis. However, other studies are needed with larger samples. The decrease in p-cresol sulfate after microbiota transfer therapy was associated with an increase in *Desulfovibrio*, a sulfate-reducing bacterium previously associated with ASD. Previous studies have found elevated levels of p-cresol in the feces and urine of autistic children. Murine models showed that p-cresol administration produces anxiety, hyperactivity, blunt interest, and stereotypic behaviors [45].

Vitamin alterations have been observed in ASD, and possibly have a role in etiology. Retinoic acid (vitamin A derivative) contributes to the development of the central nervous system through axonal elongation, neuronal differentiation, and neurite growth [46]. Retinoic acid intervenes in intestinal immunity and maintains mucosal integrity [46]. Glycoprotein CD38 expression is increased in response to retinoic acid. This results in the release modulation of oxytocin (a hormone related to social interactions) [47,48]. Our systematic search found that autistic children had increased fecal levels of 4′-apo-beta-carotenal and b,e-carotene-3,3′-diol as well as reduced retinal levels. The authors suggest that these findings may be related to impaired absorption or the conversion of plant-derived vitamin A precursor [21]. It is essential to consider that the retinal may cause damage to the nervous system, which could explain the positive correlation between retinal and the social withdrawal subscale of the Social Responsiveness Scale [21]. Complex B vitamins like thiamine-pyrophosphate, pyridoxamine, folate (B9 vitamin), and nicotinamide were altered in ASD. Decreased levels of thiamine pyrophosphate can lead to cellular damage secondary to mitochondrial dysfunction [21,49]. Pyridoxamine, a form of vitamin B6 that participates in amino acid metabolism and neural development, negatively correlates with the ABC (Autism Behavior Checklist) and CARS (Childhood Autism Rating Scale) scores [21]. This shows that complex B vitamins could be used for diagnosis and prognosis. Changes in concentrations of the cysteine methionine pathway (related to folic acid metabolism) are linked to alterations in cell proliferation, immune function, DNA synthesis, and neural development [21,50]. Nicotinamide, a form of vitamin B3 implicated in neuronal health, was a critical diagnostic metabolite with a higher concentration in the ASD group. As a result, it must be considered in future research on ASD biomarkers [4,51].

Short-chain fatty acids, acetate, butyrate, and propionate are important gut microbiota metabolites that have been associated with physiological and pathological conditions [30,52,53]. There are many bacterial groups associated with SCFA production. According to previous studies, important SCFA producers are *Bacteroides* spp. (acetate), *Bifidobacterium* spp. (acetate), *Prevotella* spp. (acetate), *Streptococcus* spp. (acetate), *Ruminococcus* spp. (acetate, propionate), *Clostridium* spp. (acetate, propionate, butyrate), *Coprococcus* spp. (propionate, butyrate), *Salmonella* spp. (propionate), *Anaerostipes* spp. (butyrate), *Eubacterium* spp. (butyrate), *Roseburia* spp. (propionate, butyrate), and *Faecalibacterium* spp. (butyrate) [31,54,55]. Of the preceding microorganisms, an increased abundance of *Bacteroides*, *Ruminococcus*, and *Faecalibacterium* has been reported in ASD. Additionally, *Streptococcus* and *Bfidobacterium* have a decreased abundance in autistic children [56]. In a case–control study, a significant enrichment of *Bacteroides*, *Akkermansia*, and *Coprococcus* was observed in children with ASD [15]. In the same study, it was also observed that ASD children were deficient in bacteria commonly associated with complex carbohydrate fermentation such as *Desulfovibrio*, *Lactobacillus*, and *Ruminococcus* [15]. The importance of bacteria with fermentation capabilities was demonstrated in a study of MTT in 18 ASD-diagnosed children who presented an improvement in behavioral and gastrointestinal symptoms, which was associated with the increment of fiber fermenters such as *Bfidobacterium*, *Prevotella*, and *Desulfovibrio* after MTT [57]. SCFAs are essential in different biological processes that could be related with ASD conditions like neurogenesis, microglia development, reduced inflammatory signaling, blood–brain barrier integrity, colonic cell energy, and maintenance of the intestinal barrier [52,53]. Constipation and other gastrointestinal symptoms can be worse with high SCFA levels [31]. Jones et al. also determined that butyric acid concentrations increased with stool looseness. This suggests that SCFAs could be used as markers of intestinal problems in ASD [29]. Autistic children frequently have gastrointestinal problems including constipation (26.17%) and diarrhea (19.92%) [58]. Considering that the difference in SCFA concentrations between autistic and non-autistic children is not always significant, more research is needed to determine the exact role of these groups of metabolites. 9-HOTrE, a fatty acid, was a key metabolite identified as a diagnosis marker in ASD by Needham et al. However, the role of this substance in autism is not known [4]

Oxidative stress and mitochondrial dysfunction have been related to ASD. For example, a lower antioxidant capacity produced by alterations in the activities of antioxidant enzymes has been observed. This predisposes to inflammation, excitotoxicity, mitochondrial alterations, and immune dysfunction in autism [59]. The decrease in the fecal concentration of the antioxidant benzaldehyde may be a sign of gut oxidative stress. Additionally, Wang et al. found an association between the higher abundance of *Eggerthella lenta* and *Clostridium botulinum* and lower concentration of benzaldehyde [22]. Arachidonic acid metabolites were also altered in the feces of autistic children and are related to oxidative stress and inflammation. This probably shows the intestinal inflammatory component seen in autism [21]. Carnitine and acetylcarnitine are markers of mitochondrial dysfunction related to oxidative stress. Acetylcarnitine and carnitine transport lipids across the mitochondrial membrane for β-oxidation. Abnormal levels of these markers are linked to reduced β-oxidation [4,60,61,62].

Dan et al. and Qureshi et al. [32,33] pointed out that metabolite combinations can discriminate between autistic and non-autistic people. This is an example of the metabolic complexity of ASD. In the future, research could consider using combinations instead of separate metabolites to better differentiate.

In the preparation of the present review, a limited number of publications on the use of intestinal microbiota metabolites as biomarkers of ASD were evident. The limited number of studies reviewed here also presented several limitations including small sample sizes; furthermore, the use of different methods for metabolic and statistical analysis made it difficult to compare the results of these reports. Available studies were mainly cross-sectional, not allowing us to determine the metabolite changes over time, which is important for prognosis analysis. The contribution of diet was not considered by all studies [23,34]. This is a potential source of bias, because nutrient consumption can modify the fecal metabolome [63]. Additionally, the selected studies here were mainly from the United States and China; therefore, data from other populations are urgently required. False discovery rate (FDR) methodologies are essential to reduce false positives in untargeted metabolomics [64]. However, Chamtouri et al., Laghi et al., and Deng et al. did not mention the utilization of these methods [23,31,34]. In each selected research, there were several possible diagnosis and prognosis molecules. It is crucial to conduct future studies to validate candidate substances as biomarkers. For this, it is necessary to determine aspects such as sensitivity, specificity, positive predictive value, negative predictive value, receiver operating characteristic (ROC) curve, cost, reproducibility, range of use, limits of detection, variability, and applicability in clinical practice [65,66]. Qureshi et al. were the only authors who reported ROC curves and sensitivity [32]. Future studies should be conducted in appropriately selected populations, with a complete metadata and a multi-omics approach to obtain results that will allow us to understand the etiology of ASD and contribute to the identification of better biomarkers [12].

In this review, we only included the analysis of gut metabolites because fecal samples are easy to obtain, could provide local information on microbiota functionality, and could be very practical in the clinic, however, we acknowledge that the intestinal metabolome does not necessarily reflect all metabolic changes associated with ASD. It is possible that metabolites present in blood, urine, saliva, and other biofluids could also be used as biomarkers for ASD.

## 5. Conclusions

This systematic review highlights the potential use of the intestinal metabolome as a source of diagnostic and prognostic biomarkers for ASD. Substances identified are associated with metabolic pathways such as amino acids, vitamins, lipids, oxidative stress, glycans, xenobiotics, and nucleotides. While the findings are promising, further studies with distinct groups of individuals with ASD around the world are needed to validate these biomarkers. Those studies should include a greater number of participants, standardized methodologies, and a longer period of follow-up. Identifying reliable biomarkers could revolutionize ASD diagnosis, prognosis, and personalized treatment strategies, ultimately improving the quality of life for children with ASD and their families. In the future, it is important to integrate metabolomic, genomic, transcriptomic and clinical data to achieve a better understanding of ASD.

## Figures and Tables

**Table 1:** Search strategies used in each database.

Database	Search Strategy
Medline	(“autism spectrum disorder” [MeSH Terms] OR autism spectrum disorder [Text Word] or ASD or neurodevelopmental disorders or asperger syndrome) and (intestinal metabolome or fecal metabolome or faecal metabolome or intestinal metabolites or fecal metabolites or faecal metabolites or colonic metabolome or colonic metabolites or colon metabolites or colon metabolites or colon metabolome or enteric metabolome or enteric metabolites or intestinal proteome or fecal proteome or faecal proteome or colonic proteome or colon proteome or enteric proteome or gut metabolome or gut proteome or gut metabolites) and (healthy children or neurotypical children or unaffected or typical or typically or control or controls) and (diagnosis or prognosis or severity) NOT (Review [Publication Type])
Web of science	(autism spectrum disorder or ASD) and (intestinal metabolome or fecal metabolome or faecal metabolome or intestinal metabolites or fecal metabolites or faecal metabolites or colonic metabolome or colonic metabolites or colon metabolites or colon metabolites or colon metabolome or enteric metabolome or enteric metabolites or intestinal proteome or fecal proteome or faecal proteome or colonic proteome or colon proteome or enteric proteome) and (healthy children or neurotypical children or control or controls) and (diagnosis or prognosis) (“autism spectrum disorder” or ASD or “neurodevelopmental disorders” or “asperger syndrome”) and (“intestinal metabolome” or “fecal metabolome” or “faecal metabolome” or “intestinal metabolites” or “fecal metabolites” or “faecal metabolites” or “colonic metabolome” or “colonic metabolites” or “colon metabolites” or “colon metabolites” or “colon metabolome” or “enteric metabolome” or “enteric metabolites” or “intestinal proteome” or “fecal proteome” or “faecal proteome” or “colonic proteome” or “colon proteome” or “enteric proteome” or “gut metabolome” or “gut proteome” or “gut metabolites”) and (“healthy children” or “neurotypical children” or unaffected or typical or typically or control or controls) and (diagnosis or prognosis or severity)
Scopus	(“autism spectrum disorder” or ASD or “neurodevelopmental disorders” or “Asperger syndrome”) and (“intestinal metabolome” or “fecal metabolome” or “faecal metabolome” or “intestinal metabolites” or “fecal metabolites” or “faecal metabolites” or “colonic metabolome” or “colonic metabolites” or “colon metabolites” or “colon metabolites” or “colon metabolome” or “enteric metabolome” or “enteric metabolites” or “intestinal proteome” or “fecal proteome” or “faecal proteome” or “colonic proteome” or “colon proteome” or “enteric proteome” or “gut metabolome” or “gut metabolites” or “gut proteome”) and (“healthy children” or “neurotypical children” or typical or typically or control or controls) and (diagnosis or prognosis or severity)
Embase	(“Autism spectrum disorder” OR ASD OR “Asperger syndrome”) AND (“intestinal metabolome” OR “gut metabolome” OR “intestinal proteome” OR “gut proteome”) (“Autism spectrum disorder” OR ASD) AND intestinal metabolome) (“Autism spectrum disorder” OR ASD) AND gut metabolome

**Table 4:** Prognostic metabolites in ASD.

Author(s)	Association with Prognosis
Needham et al. [4]	Positive correlation with ADOS Severity Scale: cystine, glycerol 3-phosphate, choline phosphate, gamma-glutamyl isoleucine, gamma-glutamyl leucine, gamma-glutamyl-alpha lysine.
Zhu et al. [21]	Elevated levels of homocysteine were negatively correlated with adaptative behavior, fine motor, personal-social behavior, and development quotient scores. High homocysteine levels had a positive correlation with inappropriate speech. Serotonin and 5-hydroxy-N-formylkynurenine levels had a negative correlation with adaptative behavior, gross motor, fine motor, personal-social behavior, and development quotient scores. N-feruloyl serotonin levels were positively correlated with Gastrointestinal Severity Index scores and with autistic mannerism and sensory alterations. 8-Hydroxy-deoxyguanosine was negatively correlated with neurodevelopment categories (adaptative behavior, fine motor and development quotient score). Vitamin B6 was slightly negatively correlated with the Autism Behavior Checklist (ABC) and Childhood Autism Rating Scale (CARS) scores. Retinal had a positive correlation with social withdrawal and CARS score. 5-Hydroxyindoleacetic acid levels were positively correlated with social motivation and CARS.
Wang et al. [22]	No metabolites associated with prognosis.
Chamtouri et al. [23]	Lower levels of histidine were significantly associated with severe symptoms of autism determined by CARS.
Kang et al. [25]	In children with ASD, microbiota transfer therapy (MTT) lowered the p-cresol sulfate levels to a concentration similar to those of the neurotypical children. The difference was significant according to an unadjusted *p*-value. 2-Hydroxycinnamate, caproate, and N-acetylmuramate had a negative correlation with the daily stool record score (based in Bristol Stool Form scale). Caproate and 2,4-dihydroxyhydrocinnamate had a negative correlation with the Gastrointestinal Symptom Rating Scale. N-Acetylmuramate had a positive correlation with the Parental Global Impressions Revised.
Jones et al. [29]	The average total SCFA concentration and acetic acid were higher in children with ASD, and these concentrations were negatively correlated with the Mullens Scale of Early Learning (MSEL) scores. However, the results were not statistically significant. Butyric acid concentrations increased with stool looseness, suggesting its concentration might be influenced by gastrointestinal conditions.
Berding et al. [30]	No metabolites associated with prognosis.
Deng et al. [31]	The results show that butyric acid was moderately negatively correlated with the SRS (Social Responsiveness Scale) and moderately positively correlated with the CSHQ (Children Sleep Habits Questionnaire).
Qureshi et al. [32]	Metabolic profile changed with treatment and became more similar in the ASD and NA groups. The ASD + GI and neurotypical carnitine concentrations were less different after microbiota transfer therapy (MTT). The average difference in normalized metabolite levels between the ASD + GI and neurotypical groups decreased significantly after MTT.
Dan et al. [33]	Hexanoic acid, chloroneb, DL-2-aminooctanoic acid, 2,5-dioxopentanoate, and desaminotyrosine related with chronic constipation.
Laghi et al. [34]	N-methylhydantoin had a higher concentration in ASD children with higher ADOS-2 scores. Aspartate, isoleucine, leucine, phenylalanine, and tyrosine were higher in children with low ADOS-2 scores. Alanine, isoleucine, leucine, methionine, phenylalanine and tyrosine had lower concentration in ASD children with Gastrointestinal Severity Index score of 4 or more. Fucose, 1,3-dihydroxyacetone characterized the highest severity group.

ASD: autism spectrum disorder; NA: non-autistic; ADOS-2: The Autism Diagnostic Observation Schedule-Second Edition; SCFA: short-chain fatty acid; ASD + GI: ASD children with gastrointestinal problems.

**Table 5:** Risk of bias of the observational studies.

Author(s)	How Large Was the Sample Size?	Were the Methods Sufficiently Described to Enable Them to Be Repeated?	Were Valid Methods Used to Determine ASD Diagnosis?	Are Sample Characteristics Clearly Described?	Are Complete Results Reported?	Were Potential Confounding Factors (Gender/Age/ASD Type or Severity) Accounted for?	Total Risk of Bias
Needham et al. [4]	2	0	0	1	0	1	4
Zhu et al. [21]	1	0	1	0	0	1	3
Wang et al. [22]	1	1	1	0	0	1	4
Chamtouri et al. [23]	2	0	0	0	0	1	3
Jones et al. [29]	2	0	0	1	0	1	4
Berding et al. [30]	2	0	2	0	0	1	5
Deng et al. [31]	2	0	1	0	0	0	3
Dan et al. [33]	1	0	1	0	1	1	4
Laghi et al. [34]	2	0	0	0	0	2	4

**Table 6:** Risk of bias of the non-randomized control trials.

Author(s)	Bias Due to Confounding	Bias in Selection of Participants into the Study	Bias in Classification of Interventions	Bias Due to Deviations from Intended Interventions	Bias Due to Missing Data	Bias in Measurement of Outcomes	Bias in Selection of the Reported Results	Overall Bias
Kang et al. [25]	Serious. Favors experimental.	Low. Toward null.	Low. Toward null.	Low. Toward null.	Low. Toward null.	Low. Toward null.	Low. Unpredictable.	Low. Toward null.
Qureshi et al. [32]	Serious. Favors experimental.	Low. Toward null.	Low. Unpredictable	Low. Toward null.	Low. Toward null.	Low. Toward null.	Low. Toward null.	Low. Toward null.

## Data Availability

No new data were created or analyzed in this study. Data sharing is not applicable to this article.

## Supplementary Materials

The following supporting information can be downloaded at: https://www.mdpi.com/article/10.3390/metabo15040213/s1, Table S1: Excluded studies.

## Abbreviations

The following abbreviations are used in this manuscript:

ABC	Autism Behavior Checklist
ADI-R	Autism Diagnostic Interview-Revised
ADOS-2	Autism Diagnostic Observation Schedule, Second Edition
ASD	Autism spectrum disorder
CARS	Childhood Autism Rating Scale
DSM-5	Diagnostic and Statistical Manual of Mental Disorders, Fifth Edition
GABA	Gamma-aminobutyric acid
GC	Gas chromatography
GC-MS	Gas chromatography coupled with mass spectrometry
GI	Gastrointestinal
H-NMR	Proton nuclear magnetic resonance
LC–MS	Liquid chromatography-mass spectrometry
LC–MS/MS	Liquid chromatography-tandem mass spectrometry
MTT	Microbiota transfer therapy
NA	Non-autistic
OFM	Optimized fecal models
PRISMA	Preferred Reporting Items for Systematic Reviews and Meta-Analysis
ROBINS-I	Risk Of Bias In Non-Randomized Studies of Interventions
SCFA	Short-chain fatty acids.
UHPLC	Ultra-high performance liquid chromatography
UHPLC-MS/MS	Ultra-high performance liquid chromatography-tandem mass spectroscopy.
UHPLC-QE	Ultra-high performance liquid chromatography coupled with quadrupole electrospray ionization mass spectrometry
