# Supplementary material for: Intestinal Metabolome for Diagnosing and Prognosing Autism Spectrum Disorder in Children: A Systematic Review
Source: Metabolites. 2025 Mar 21;15(4):213. doi: 10.3390/metabo15040213 (PMC12029135; doi:10.3390/metabo15040213)
Supplement: Supplementary file 1 [file metabolites-15-00213-s001.zip › metabolites-3522471-supplementary.pdf]

Supplementary table 1. Excluded studies.

Author(s)	Year	Title	Reasons for exclusion
Yin et al.	2020	A Comparative Evaluation of Tools to Predict Metabolite Profiles from Microbiome Sequencing Data	Compares tools to predict metabolite profile.
Olesova et al.	2020	A Novel UHPLC-MS Method Targeting Urinary Metabolomic Markers for Autism Spectrum Disorder	Analyzes urinary metabolome
Piras et al.	2020	Alteration of the Intestinal Permeability Are Reflected by Changes in the Urine Metabolome of Young Autistic Children: Preliminary Results	Analyzes urinary metabolome. Authors found intestinal permeability in 3 participants.
de Angelis et al.	2015	Autism spectrum disorders and intestinal microbiota	Review
Kang et al.	2018	Differences in fecal microbial metabolites and microbiota of children with autism spectrum disorders	No accepted diagnosis method. Children were assessed with Autism Treatment Evaluation Checklist (ATEC) and Pervasive Developmental Disorder Behavior Inventory (PDD-BI)
Troisi et al.	2020	Genome, environment, microbiome and metabolome in autism (GEMMA) study design: Biomarkers identification for precision treatment and primary prevention of autism spectrum disorders by an integrated multi-omics systems biology approach	Study protocol without results.
Mu et al.	2020	Metabolic Framework for the Improvement of Autism Spectrum Disorders by a Modified Ketogenic Diet: A Pilot Study	Analyzes plasma metabolome
Ming et al.	2012	Metabolic perturbance in autism spectrum disorders: A metabolomics study	Analyzes urinary metabolome
Qureshi et al.	2022	Multivariate Analysis of Metabolomic and Nutritional Profiles among Children with Autism Spectrum Disorder	Analyzes urinary and blood metabolome
Quan et al.	2020	Plasma trimethylamine N-oxide, a gut microbe-generated phosphatidylcholine metabolite, is associated with autism spectrum disorders	Analyzes blood metabolome
Osredkar et al.	2023	Relationship between Excreted Uremic Toxins and Degree of Disorder of Children with ASD	Analyzes urinary metabolome
Noto et al.	2014	The urinary metabolomics profile of an Italian autistic children population and their unaffected siblings	Analyzes urinary metabolome
Qureshi et al.	2023	Towards the development of a diagnostic test for autism spectrum disorder: Big data meets metabolomics	Analyzes plasma metabolome
Daneberga et al.	2022	Urinary organic acids spectra in children with altered gut microbiota composition and autistic spectrum disorder	Analyzes intestinal microbiome and urinary metabolome
